# Delayed double reading of whole blood clotting test (WBCT) results at 20 and 30 minutes enhances diagnosis and treatment of viper envenomation

**DOI:** 10.1186/s40409-018-0151-1

**Published:** 2018-05-16

**Authors:** Jordan Max Benjamin, Jean-Philippe Chippaux, Bio Tamou Sambo, Achille Massougbodji

**Affiliations:** 1 Center for the Study and Research of Malaria Associated with Pregnancy and Childhood (CERPAGE), 08 BP 841 Cotonou, Bénin; 2 Whitman College, Department of Biology, Walla Walla, WA 99362 USA; 3 IRD UMR216, Mère et enfant face aux infections tropicales, 75006 Paris, France; 4 PRES Sorbonne Paris Cité, Université Paris Descartes, Faculté de Pharmacie, 75270 Paris, France; 5 Département de chirurgie et spécialités, Faculté de Médecine, Université Parakou, Parakou, Bénin

**Keywords:** Africa, Snakebite, *Echis*, Envenomation, Whole blood clotting test, WBCT, Venom-induced consumption coagulopathy, Carpet viper, Saw-scaled viper

## Abstract

**Background:**

The whole blood clotting test (WBCT) is a simple test of coagulation that is often used in the assessment, diagnosis, and therapeutic monitoring of snakebite patients in sub-Saharan Africa. WBCT requires only a clean glass tube and several milliliters of venous blood and is ideal for use in poorly equipped health centers throughout the rural areas where 95% of snakebites occur. However, questions surrounding the accuracy and reliability of the test remain unanswered due to variations in testing conditions and a lack of comparative research with which to validate them. This is the first study to evaluate WBCT results at both 20-min (WBCT20) and 30-min (WBCT30) reading times in the same group of snakebite patients.

**Methods:**

In order to define the best reading time, the authors compared the results of serial WBCT evaluation at both 20 and 30 min after collection in 23 patients treated for snake envenomation in Bembèrèkè, northern Benin.

**Results:**

WBCT results were identical at both reading times in patients without coagulopathy or when coagulation was restored permanently following a single dose of antivenom. Out of 17 patients with coagulopathy, 14 showed discrepancies between WBCT20 and WBCT30 results in at least one pair of serial evaluations. These could be completely contradictory results (e.g. normal clot at WBCT20 and no clot at WBCT30) or a marked difference in the quality of the clot (e.g. no clotting activity at WBCT20 and an unstable partial clot at WBCT30). WBCT discrepancies were encountered most frequently in three situations: initial normalization of hemostasis following antivenom therapy, detection of a secondary resumption of coagulopathy, or final restoration of hemostasis after a secondary resumption had occurred.

**Conclusions:**

This study suggests that the WBCT is robust and that a sequential reading should improve the diagnosis and monitoring of venom-induced coagulopathies. It also indicates the possibility of discrepancies in the sensitivity of WBCT20 and WBCT30 for detecting the resolution or reoccurrence of coagulopathy and identifies how these findings, if confirmed, may be used to increase the efficacy and efficiency of antivenom treatment in the field.

## Background

In developing countries, particularly in sub-Saharan Africa, most envenomations occur in rural areas and are managed in peripheral health centers lacking the capability to perform automated laboratory tests for diagnosis or monitoring of envenomed patients [1, 2]. Venom-induced consumption coagulopathies (VICC) are present in more than two-thirds of African snake envenomations [3]. When used correctly, the whole blood clotting test (WBCT) is a simple, effective, affordable bedside examination that provides valuable information during initial assessment and ongoing management of snakebite patients throughout the course of treatment [4–7]. Use of the WBCT for detecting VICC in snakebite patients was standardized by Sano-Martins et al. [8]. It requires collection of a small amount of venous blood (about 2 mL) in a dry and clean glass tube to evaluate the coagulation time by simple direct observation of clot formation and stability 20 min after collection. Several field studies have confirmed that the WBCT was sufficiently sensitive and specific to be of clinical value [4, 5, 9–11]. It is a comprehensive blood test to diagnose and monitor coagulopathy in patients bitten by most viper species, which is valid throughout the world. It is also useful for diagnosis of coagulopathy in patients bitten by dangerous colubrids and many of the elapid species found in Australasia. The WBCT is particularly useful when the symptoms are mild or before the onset of clinical hemorrhagic syndrome [5, 6, 9, 12]. In addition, the WBCT is an important criterion for evaluating the efficacy of antivenom therapy [3, 9, 13, 14].

The test should be carried out in a clean, dry, glass tube completely free of detergent. Deviations from this protocol such as the use of poorly rinsed glassware or any non-glass tubes can alter the outcome of the test and lead to inaccurate interpretation [15]. In addition, there have been reported contradictory results regarding accuracy of the WBCT under varying conditions [6, 16, 17]. It is generally recommended to observe the clot at the 20th minute (WBCT20), but many studies have reported a delay in reading until the 30th minute (WBCT30). This likely results from the many tasks that must be accomplished by clinicians during initial assessment and stabilization of the patient, which oftentimes takes place in overcrowded and understaffed rural health centers [13]. This reading delay is likely to affect the test results but has never been evaluated [16].

In order to see how the WBCT performs in a realistically challenging clinical setting, we performed a preliminary study in 23 snakebite patients treated in rural Beninese hospital in order to compare the results of WBCT interpretation at 20 and 30 min and assess the reliability of the measurements taken at different time points from the same group of patients.

## Methods

Twenty-three snakebite patients were evaluated using both WBCT20 and WBCT30 readings at the Hôpital Evangelique de Bembèrèkè in Bembèrèkè, northern Benin, between late June and early October 2012. In order to accurately reflect the challenging circumstances in which WBCT testing is used in rural Africa, all patients who were evaluated using both WBCT20 and WBCT30 throughout the course of care were included in this study regardless of treatment delay and severity of envenomation. Assessment, diagnosis, treatment, and clinical management of all patients from intake to discharge was performed by one of the authors (JB) to ensure homogeneity of testing methods and results. Snake identifications were made by JB based on examination of the snake or description of the snake by the patient or family in conjunction with the clinical syndrome of envenomation. Clinical examination and WBCT interpretation were performed during the initial assessment at hour 0 (H_0_) in all patients prior to administration of the polyvalent antivenom (Antivipmyn® Africa, Bioclon, Mexico). The WBCT was repeated during serial reexaminations at H_3_, H_6_, H_12_, H_24_ and every 24 h thereafter until permanent restoration of blood clotting (no hemorrhage or coagulopathy for more than 48 h) and complete resolution of the envenomation had been achieved.

**Fig. 1:**
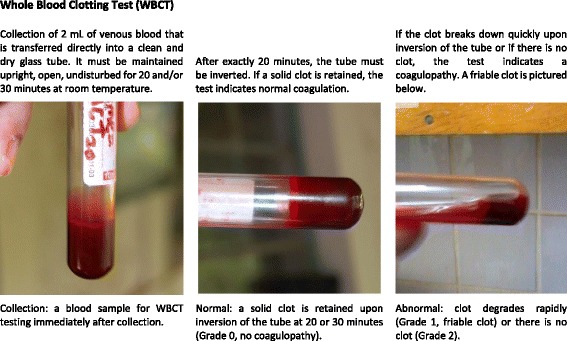
Collection and evaluation of the whole blood clotting test (WBCT). The WBCT20 should be taken from every suspected envenomation patient in Africa. The test must be performed in a clean dry, glass tube free of preservatives, soaps, or other contaminants – even plain vacutainers must be precleaned on site. A 10-mL glass test tube is well suited for this test. Measure out 2 mL into each tube, and when collecting from existing intravenous (IV) catheters perform a 2 mL waste draw to ensure collection of an undiluted sample. Assess at 20 and 30 min precisely. Partial clots and clots that rapidly degrade upon examination count as abnormal; this usually occurs within the first 5–30 sec after inversion of the tube. Solid clots that remain intact are considered normal. Test against blood from a healthy donor if results seem inconsistent with the clinical picture. The same tube may be used for both tests as long as it is not disturbed in between readings at 20 and 30 min. Note that the labeled vacutainer in the first two images was used to produce these photos for educational purposes and that vacutainers were not used for specimen collection during the study. The abnormal sample shown in the third picture was collected 3 h after antivenom administration in a patient suffering from an *E. ocellatus* envenomation during the study

The WBCT was always evaluated twice in all patients: first in the 20th minute after collection and again at the 30th minute (Fig. 1). The following grading scale was used for WBCT interpretation: Grade 0: normal coagulation (solid, stable clot); Grade 1: abnormal coagulation (unstable or friable clot that disintegrates rapidly upon inversion of the test tube); Grade 2: no coagulation (Table 1).

**Table 1:** Grading system used to score WBCT results at both reading times

	Normal or abnormal?	Visual appearance
Grade 0	Normal coagulation	Stable, solid clot that maintains its shape and clings to the glass vessel without issues after rotation of the tube.
Grade 1	Abnormal, but there is some clotting activity apparent at the 20^th^ and 30^th^ minutes	There is some blood in solid form, but it fails to adhere to the glass as a single plug and either disintegrates completely (friable clot) or partially degrades ≤30 sec after rotation.
Grade 2	Abnormal with no clotting activity apparent at the 20^th^ and 30^th ^minutes	The entire blood sample remains in a free-flowing liquid state with no demonstrable clotting whatsoever upon rotation. This is immediately apparent during rotation of the tube.

All tests were timed to ensure that the readings occurred precisely at the 20th and 30th minute after collection. Most tests were performed using a separate tube for each reading time, particularly those performed in the first 24 h, but we observed no discrepancies between the two methods and in some cases it was easier to perform the test in a single tube due to the circumstances (multiple critically ill patients at one time, quantity of blood collected, etc.). When a single tube was used, it was tipped at both 20 and 30 min without disturbing it between readings. When two tubes were used, the WBCT20 tube was tipped a second time at 30 min and invariably yielded the same results as the WBCT30 tube which was only tipped at 30 min. WBCT samples were retained for observation in the glass tube for at least 24 h before disposal. Ambient temperature in the wards where tests were performed typically ranged from 24 to 27 °C.

It is widely accepted that the test should be conducted in a clean, dry, glass tube free of any contaminants such as soap residue. However, there are no tubes produced specifically for the WBCT and it is generally performed using whatever clean glass vessels (test tubes, medication vials or ampoules, etc.) are available in the clinic at that time. In order to ensure that the results were both accurate and applicable under realistic conditions, we conducted an informal test at the beginning of the study using various appropriate-sized containers that were available at the hospital.

Blood samples were collected from five healthy volunteers and evaluated in:

- disposable BGL Sysmet 10 mL plain glass red top vacutubes, 13 × 100 mm;
- standard disposable plastic syringes, between 3 and 10 mL;
- screw cap clear glass medication vials, approximately 19 × 73 mm;
- 5-mL clear glass serum bottles, 23 × 47 mm; and
- an assortment of reusable 10 mL plain glass test tubes, approximately 13 × 100 mm, that were maintained by clinical laboratory personnel for the WBCT and a number of other blood tests.

**Fig. 2:**
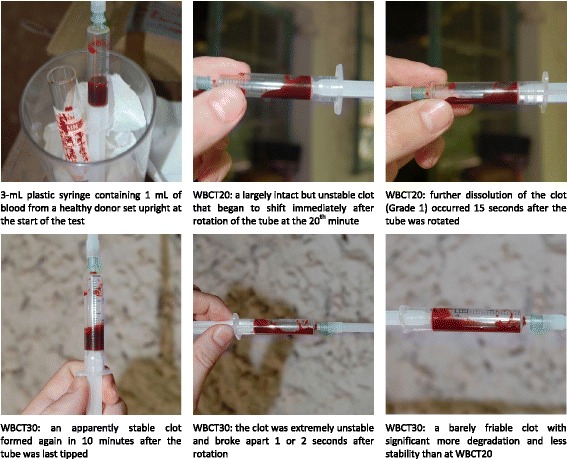
Blood from healthy donors failed to clot normally in plastic syringes at both reading times

Blood failed to clot normally in plastic syringes at both reading times (Fig. 2) and results in the BGL Sysmet vacutubes were inconsistent. The glassware from groups (c), (d) and (e) yielded accurate results in all donors. The clots in these tubes remained intact when disposed of 3 h later, indicating the absence of any physiological hyperfibrinolysis in the control group. The five donors consisted of two males and three females between 21 and 30 years of age. They were all visiting healthcare workers from Europe, Canada, and the United States with known medical histories and without medications or conditions that could interfere with normal coagulation. All WBCT samples in the formal study were evaluated using the plain glass 10-mL test tubes from group (e) and all tubes were carefully examined by JB prior to use to ensure that they were free of any scratches, residues, or other imperfections that could potentially interfere with the test. Pre-checked tubes were placed in plastic bags and set aside in a designated snakebite kit to ensure they would not be exchanged or contaminated prior to use. When necessary, tests were repeated in fresh tubes to confirm results that were inconsistent with the clinical picture at the time.

Blood samples for WBCT testing were drawn from the peripheral IV catheter during initial cannulation at H_0_, through venipuncture in the opposite arm during the first 24 h after an antivenom treatment, and drawn by either method thereafter. Venipuncture was used whenever possible and comprised the majority of samples. In all occasions when it was necessary to obtain samples from existing peripheral IV catheters, a 2 mL waste draw was performed first to ensure collection of an undiluted sample. Numerous studies have validated this as an acceptable alternative to venipuncture with no statistically significant difference in hematocrit, hemoglobin, CBC, PT/INR, aPTT and other parameters between the two methods [18–21].

Antivipmyn® Africa (AA) dosage was determined by the severity of the individual envenomation, which was assessed using a clinical gradation scale of local and systemic findings given elsewhere as well as hematocrit testing by centrifugation to detect anemia [3, 9, 10, 13]. The extent of edema and bleeding was scored according to the chart detailed below (Table 2). Patients with incoagulable blood (defined by abnormal WBCT results at 20 and/or 30 min) and/or abnormal atraumatic bleeding (as described in Table 2) at H_0_ received an initial antivenom dose of two vials in 10 mL of sterile water administered by direct intravenous push at a flow rate of 2 mL/min. Those with pain and edema but without coagulopathy and/or bleeding received a single vial of antivenom at H_0_. Additional doses of two vials were given in the event of persistence or resumption of bleeding at any the serial reassessment points listed earlier. IV catheters were flushed with 10 mL of sterile water or isotonic saline immediately following antivenom administration.

**Table 2:** Clinical progression of edema and bleeding for viper envenomations in Benin

	Edema	Bleeding
Stage 1	Does not extend beyond wrist/ankle	Persistent atraumatic bleeding from the bite wound >1 h
Stage 2	Does not extend beyond major joints (elbow/knee)	Bleeding from old cuts and wounds elsewhere on patient
Stage 3	Extends beyond major joints	Spontaneous bleeding from healthy mucosa (i.e. gingiva)
Stage 4	Reaches but does not extend beyond multiaxial joints (shoulder/hip)	Externalization of internal bleeding (hematemesis, melena, etc.)
Stage 5	Extensive edema beyond multiaxial joints	Cerebral, meningeal, intra-abdominal, or retroperitoneal hemorrhage; critical hemorrhagic shock

## Results

The group of 23 patients consisted of 15 envenomations by *E. ocellatus*, three by *B. arietans*, three by *Naja nigricollis* or *N. katiensis*, one dry bite of unknown origin and one peculiar dry bite or very mild envenomation from an unidentified rear-fanged colubrid or small elapid. With the exception of the two suspected dry bites, all patients were treated with 1–6 vials of Antivipmyn® Africa; there were no deaths and all patients recovered without major sequelae. There were 17 patients (74%) who showed clinical signs of abnormal local or systemic bleeding and abnormal WBCT (Table 3) meeting diagnostic criteria for the hemorrhagic syndrome.

**Table 3:** Description, characteristics, and response to initial antivenom therapy in 17 patients with abnormal WBCT results

Case n.	WBCT20 ≠ WBCT30	Sex	Age	Snake species	Delay (hours)	Stage of edema/bleeding	HCT at H_0_ (%)	N. vials AA at H_a_	Initial WBCT20 at H_0_	Initial WBCT30 at H_0_	1^st^ Normal WBCT20 at H_a_	1^st^ Normal WBCT30 at H_a_	External bleeding arrested at H_a_
1	Group 3a Group 4	M	7	*Echis*	72	E5, B4	24.4	H_0_ = 2 H_3_ = 2	No clot	No clot	H_3_	H_3_	H_6_
2	Group 2	M	9	*Echis*	12	E3, B5	13.0	H_0_ = 2	No clot	No clot	H_24_	H_48_	H_3_
3	Group 2 Group 3b	M	7	*Echis*	24	E2, B3	40.0	H_0_ = 2	No clot	No clot	H_12_	H_6_	H_3_
4	Group 1	M	42	*Bitis*	20	E5, B1	31.0	H_0_ = 2	Weakly friable	Improved friable	H_3_	H_3_	H_3_
5	Group 2 Group 4	M	15	*Echis*	48	E5, B5	14.0	H_0_ = 2 H_6_ = 2	No clot	No clot	H_3_	H_6_	H_12_
6		M	25	*Echis*	192	E2, B4	31.6	H_0_ = 2	No clot	No clot	H_3_	H_3_	H_3_
8		M	41	*Echis*	120	E1, B4	42.0	H_0_ = 2	No clot	No clot	H_3_	H_3_	H_3_
10	Group 3a Group 4	M	18	*Echis*	96	E2, B5	21.0	H_0_ = 2 H_3_ = 2	No clot	No clot	H_6_	H_6_	H_12_
12	Group 3a	M	25	*Echis*	1	E1, B3	35.0	H_0_ = 2 H_3_ = 2	No clot	No clot	H_6_	H_6_	H_6_
13	Group 2	M	20	*Echis*	72	E3, B4	36.0	H_0_ = 2	No clot	No clot	H_3_	H_24_	H_3_
^a^15	Group 1	F	18	*Bitis*	72–168	E1, B1	32.3	H_0_ = 1	Friable	Normal	H_3_	N/A	Before arrival
^b^17	Group 1	M	30	*Echis*	120	E2, B5	39.0	H_0_ = 2	Friable	No clot	H_3_	H_3_	Internal only
18		M	25	*Echis*	4	E2, B3	N/A	H_0_ = 2	No clot	No clot	H_3_	H_3_	H_3_
19	Group 2	F	15	*Echis*	72	E3, B5	24.0	H_0_ = 2	No clot	No clot	H_3_	H_6_	H_3_
20	Group 2	M	35	*Echis*	3	E1, B4	36.0	H_0_ = 2 H_3_ = 2	No clot	No clot	H_24_	H_72_	H_6_
^c^22	Group 3b	F	12	*Echis*	24	E2, B5	11.0	H_0_ = 2	No clot	No clot	H_6_	H_6_	H_3_
23	Group 2	M	22	*Echis*	69	E2, B5	11.0	H_0_ = 2	No clot	No clot	H_6_	H_24_	H_3_

^a^15 = Patient alternately stated that the bite occurred either 3 or 7 days earlier; abnormal local bleeding reportedly began 48 h after the bite but resolved shortly before she came to the hospital. Freshly dried blood was visible around the bite site

^b^17 = Hematemesis and gingival bleeding resolved shortly before arrival, but he presented with gross hematuria and signs of subarachnoid hemorrhage (widening pulse pressure with systolic hypertension; bradycardia; irregular breathing; altered mental status). Signs of increased intracranial pressure resolved between H_12_ and H_24_. There was still gross hematuria when the patient discharged himself against medical advice at H_144_, but the overall clinical picture suggested that kidney injury rather than active venom was causing the issue at the time of discharge

^c^22 = Gingival bleeding stopped after antivenom at H_0_. No WBCT taken at H_3_, but WBCT20 and WBCT30 were both normal at H_6_

The 17 patients with hemorrhagic envenomations included two *B. arietans* envenomations and 15 *E. ocellatus* envenomations. Most (11/17, 65%) showed anemia (hematocrit ≤35%) and nearly half of these patients (7/17, 41%) arrived in critical condition with a late-stage hemorrhagic syndrome (stages 4 or 5), unstable vital signs, major complications, and a high likelihood of imminent death unless urgent interventions were taken. The findings of the initial clinical assessment at H_0_ (abnormal local or systemic bleeding, anemia, blistering, necrosis, edema, etc.) supported the results of the initial diagnostic WBCT in all of our patients, and there were no false positive results at H_0_. The remaining 6/23 patients without coagulopathy or bleeding are detailed below (Table 4) and included four clinically significant envenomations that received antivenom treatment and two suspected dry bites that were managed symptomatically.

**Table 4:** Description, clinical findings, and treatment of six patients without coagulopathy or bleeding

Case n.	Sex	Age (year or months)	Snake	Delay	Edema (stage)	Pain	Blistering/necrosis	Severity	N. vials AA at H_a_
7	M	12 y	^a^ *Naja*	72 h	2	Yes	None	Moderate	H_0_ = 1
9	M	24 y	^b^ *Naja*	72 h	2	Yes	None	Mild	H_0_ = 1
11	M	20 y	Unknown	3 h	0	No	None	Mild or dry bite	None
14	F	10 m	*Bitis*	96 h	5	Yes	Extensive, superficial	Severe	H_0_ = 1
16	F	25 y	^c^ *Naja*	17 days	3	Yes	Profound necrosis	Moderate	H_0_ = 1
21	M	20 y	Unknown	3.5 h	0	No	None	Dry bite	None

^a, b, c^ = Suspected based on clinical presentation, circumstances of the bite, and description of both the snake and the symptoms experienced in the first 48 h after the bite (dyspnea, weakness, paresthesia, neuropathic pain, etc)

Discordance in WBCT results at 20 and 30 min was observed in 82% (14/17) of patients with bleeding disorders who received both tests, and are detailed in Table 3. The variation we observed between WBCT results at 20 versus 30 min-reading times was substantial, with an average time to resolution of coagulopathy following antivenom treatment of 6.7 h by WBCT20 and 13.1 h by WBCT30. Discrepancies were noted in at least one pair of serial WBCT readings and were identified specifically in four circumstances: upon initial assessment at H_0_ (Group 1, *n* = 3); upon initial normalization of hemostasis following antivenom therapy (Group 2, *n* = 7), upon secondary resumption of coagulopathy (Group 3a, *n* = 3); and upon restoration of hemostasis after a secondary resumption of coagulopathy (Group 3b, *n* = 2). There were three additional cases in which inconsistency was noted between results of the WBCT and the clinical assessment of the patient (Group 4). In these cases, both the WBCT20 and WBCT30 indicated restoration of hemostasis despite the presence of ongoing external and internal bleeding, which had markedly improved with antivenom therapy but persisted solely at the site of the gingival sulci for an additional 3 to 6 h before complete cessation of bleeding was observed. Discrepancy between results of WBCT readings was observed in only one of these three patients (case no. 5): WBCT20 was restored at H_3_, WBCT30 at H_6_, and bleeding resolved at H_12_. Tests were repeated using a new set of tubes in all three cases to confirm the peculiar results. Finally, in the remaining 3/17 patients (case no. 6, 8, and 18) normal coagulation was restored permanently and completely by H_3_ following a single dose of antivenom at H_0_.

Differences noted during initial assessment (Group 1) presented as variations in severity of coagulopathy (one gradation of WBCT scoring on the scale detailed in methods) rather than entirely contradictory results; two patients (case no. 4 and 15) exhibited a coagulopathy that improved by one grade between 20 and 30 min and the third patient (case no. 17) had a friable clot at 20 min that was completely incoagulable by 30. In six of seven cases, the two tests did not normalize simultaneously, WBCT20 was corrected earlier than WBCT30. Resumption of coagulopathy was observed in a total of seven patients with *E. ocellatus* envenomations and five of them exhibited discrepancies in WBCT results (Groups 3a and 3b; Table 5).

**Table 5:** Resumption and resolution of coagulopathy by WBCT20 and WBCT30 in seven patients with *Echis* envenomations

Case No.	^a^Discrepancies in secondary events	Secondary resumption –WBCT20 at H_a_	Secondary resumption –WBCT30 at H_a_	Recurrence of symptoms	Additional AA given	Final control WBCT20 at H_a_	Final control WBCT30 at H_a_	Resolution of symptoms
1	Initial detection (Group 3a)	Remained normal	H_6_	No, patient improving	No	H_3,_ no resumption	H_24_	H_6_
2	No	H_72_	H_72_	Diffuse pain and headache	2 vials at H_72_	H_120_	H_120_	≤ H_96_
^a^3	Final resolution (Group 3b)	H_72_	H_72_	Edema and blistering	No, due to H_0_ anaphylaxis	H_144_	H_192_	H_192_
^b^10	Initial detection (Group 3a)	H_48_	H_27_	Internal bleeding	2 vials at H_48_	H_120_	H_120_	≤ H_72_
12	Initial detection (Group 3a)	Remained normal	H_24_	No, patient improving	No	H_6_, no resumption	H_48_	H_6_
^c^19	No	H_24_	H_24_	Gingival bleeding	2 vials at H_24_	H_72_	H_72_	H_27_
^d^22	Transient resolution (Group 3b)	H_96_ and H_168_	H_96_	Internal bleeding	2 vials at H_120_ 2 vials at H_168_	H_240_	H_240_	H_192_

^a^3 = We elected to manage the secondary recurrence of envenomation symptomatically for as long as possible because the patient had already experienced two separate anaphylactoid reactions and there were very limited resources on hand for resuscitating a pediatric cardiac arrest

^b^10 = The patient presented with a subarachnoid hemorrhage at H_0_ which initially resolved by H_19_, but signs and symptoms returned at H_27_ in conjunction with a recurrence of coagulopathy by WBCT30. At H_48_, his WBCT20 also turned positive. He was treated with additional two vials of antivenom at this time and made a full recovery without sequelae

^c^19 = Gingival bleeding resumed at H_24_ after initially resolving at H_6_

^d^22 = Two separate resumptions occurred with this patient. Resumption of WBCT20 and WBCT30 was recorded at H_96_, but most likely began around H_72_ when signs of intraperitoneal hemorrhage began to occur. The test was normal at H_48_, but could not be collected at H_72_

**Fig. 3:**
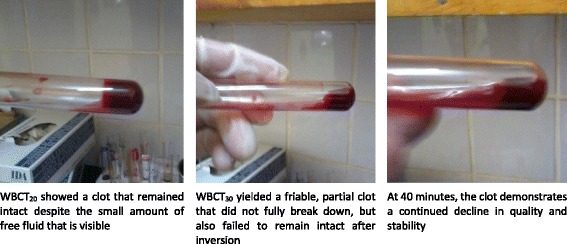
Discrepancy in WBCT results at 20 and 30 minutes in a patient with an *Echis ocellatus* envenomation. Transient, asymptomatic resumption of coagulopathy at H24 by WBCT30 alone. This patient (case no. 12) presented to the hospital less than 1 h after an *Echis ocellatus* bite to the 5^th^ finger of his right hand and was already experiencing completely incoagulable blood (Grade 2) at both reading times despite the absence of any bleeding from the bite site or elsewhere. Two vials of antivenom were administered at H_0_ and additional two vials were given at H_3_ when assessment revealed a new onset of gingival hemorrhage in addition to persistence of abnormal WBCT results. Bleeding ceased within an hour and WBCT20/WBCT30 were both normalized during the next evaluation at H_6_. There was a brief resumption of coagulopathy by WBCT30 at H_24_ that was inconsistent with the overall picture of clinical improvement and resolved 24 h later at H_48_. WBCT20 was restored permanently at H_6_

In two patients (cases no. 1 and 12, Fig. 3) there was a transient resumption of WBCT30 in the first 24 h that was inconsistent with the clinical picture of improvement observed at the time of the test, and there was no resumption of WBCT20 in either case. In the other five patients, a resumption of coagulopathy coincided with a resumption of envenomation signs and symptoms (gingival bleeding, internal bleeding, pain, edema, etc.) and therefore fit the diagnosis of a recurrent envenomation. In one of these patients (case no. 10), WBCT30 detected the recurrence of envenomation 21 h before it was detected by WBCT20 and coincided with the earliest symptoms of a renewed subarachnoid hemorrhage.

In the case of discrepancies upon final restoration of hemostasis after a secondary resumption of coagulopathy, WBCT20 was the first test to be corrected. In one of these cases, the improvement at WBCT20 corresponded with improvement in the recurrent envenomation. In the other case, the test remained abnormal at both reading times until WBCT20 briefly resolved at H_144_, which was inconsistent with the clinical picture of an active envenomation. WBCT20 became abnormal once again 24 h later at H_168._ Bleeding stopped and hematocrit stabilized ≤24 h after the final dose of antivenom was given at H_168_, but WBCT20 and WBCT30 remained abnormal until H_240_.

The ambient temperature in rooms where WBCT testing was performed was between 24 and 27 °C. Whether or not these temperature ranges influenced the coagulation time remains unknown. However, the consistency of temperatures during the study lends some uniformity to the results and the methods used here are consistent with those employed at rural healthcare facilities throughout Africa.

## Discussion

The frequency of coagulation disorders caused by the bite of *Echis ocellatus* (>75% of snakebite patients presenting to the hospital in Bembéréké) is corroborated by previously published observations in this region of Benin [3, 22–25]. The small number of patients is compensated by the homogeneity of measurements of the WBCT, all performed by the same clinician (JB) according to standard protocols, and of the species of snake responsible for the envenomation. In our series, the overall agreement between WBCT20 and WBCT30 was 100% in the absence of bleeding disorders or after they had resolved clinically. This suggests that the WBCT is likely to be a useful and clinically applicable test despite limitations and concerns over its incorrect application [16]. The majority of discrepancies between WBCT results were observed after the initiation of antivenom therapy and during the period before recovery – a poorly understood area of interaction between the venom, the antivenom, and the unique characteristics of the patient. Differences between WBCT readings were most often seen upon initial resolution of coagulopathy or after secondary resumptions of coagulopathy, as determined by WBCT. The vast majority of discrepancies observed did not appear to result from a failure of the test because they did not occur when coagulopathy was either active (prior to treatment) or non-existent and, secondly, because the resumption in these cases was linked to the presence of clinical or hematological instability. Discrepancies seemed therefore to result from the action of the venom on blood coagulation, the efficacy of antivenom treatment, or the patient’s individual response to both the treatment and the envenomation.

In two patients, the blood did not coagulate at 20 min, but did 10 min later, suggesting either that a quantitative or qualitative deficiency of coagulation factor was responsible for the clotting delay or that there was an error in the collection method. One of these patients exhibited abnormal coagulation and persistent bleeding from the bitten limb 20 h after he was bitten twice by a large 2-m *B. arietans*. The dead snake in this case was taken to the hospital by the patient and identified by a herpetologist (JB). In contrast to the consumption coagulopathies seen in *E. ocellatus* envenomations, the patient showed a delayed coagulation with a very weak friable clot at 20 min, a markedly improved (but still friable) clot at 30 min, and a stable clot at 50 min that remained intact until disposal 24 h later. A second patient exhibited similarly delayed coagulation with a partial clot observed at 20 min and a stable clot at 30 min following a significantly less severe envenomation from a juvenile *B. arietans* that occurred at least 72 h earlier. She did not present with active bleeding, but reported that the wound started to bleed again 48 h prior to arrival and stopped several hours before she came to the hospital, which was supported by dried blood on and around the bite site in the absence of any apparent trauma.

Permanent restoration of hemostasis and cessation of bleeding in both patients was achieved at H_3_ following antivenom administration, suggesting that the findings may indeed have resulted from something related to the envenomation rather than the WBCT collection. Clinical coagulopathy due to envenomation by *B. arietans* is uncommon, but has been reported previously. Prolonged activated partial thromboplastin time and prothrombin time were reported in a patient who presented with systemic bleeding after *B. arietans* envenomation in Senegal [26]. Spontaneous systemic bleeding, thrombocytopenia, and anemia have been reported in three patients from northern Nigeria despite normal WBCT20 results and normal levels of clotting factors and fibrin degradation products (FDPs) [27]. WBCT30 was not reported in these cases.

In patients with coagulopathies due to *E. ocellatus* envenomation, we observed that any clots formed at 20 or 30 min during the first 24 h had often degraded again within 60 min of collection and returned to an incoagulable state prior to disposal. Further observation of blood samples collected from *E. ocellatus* envenomation patients in this study revealed the apparent formation and subsequent degradation of multiple clots in quick succession during the first several minutes after collection. Qualitative observation of the tubes during this time indicated a progressive reduction of this activity over time and by 15 min after collection the blood sample was approaching the state it would maintain upon evaluation at 20 min. When permanent resolution of hemostasis was achieved, stable clots generally persisted in the tube without any significant retraction and remained in this state 24 h later during disposal.

In six out of the seven patients from Group 2 with discrepancies in the initial resolution of coagulopathy, blood clotted within 20 min then returned to liquid form 10 min later. This situation suggests hyperfibrinolysis that could be due to either a factor present in the venom that was not neutralized by the antivenom, or a physiological factor present in the patient’s plasma. The presence of abnormal bleeding in the absence of coagulopathy reported in the three patients from Group 4 is therefore perplexing, but not entirely unprecedented. Authors of Nigerian cases suggest that bleeding may have resulted from a combination of direct action of the venom on the vascular endothelium in conjunction with a diminished platelet count [27]. All three of our cases involved young patients with severe *E. ocellatus* envenomation, significant treatment delays, severe anemia, and late-stage bleeding syndromes. A complete blood count performed on the youngest patient found leukocyte count of 10.1 × 10^3^/μL, erythrocyte count of 3.28 × 10^6^/ μL, hemoglobin of 8.5 g/dL, and platelets of 229 × 10^3^/μL. Whether the unusual bleeding resulted from the physiology of the patient, the venom, or the sampling method is impossible to determine with the available information.

Unfortunately, it has not been possible to continue hematological investigations in these patients because of the lack of appropriate laboratory facilities or more comprehensive testing capabilities on site. This issue, however, illustrates the importance of the WBCT in the rural healthcare centers where it is often the only means of detecting coagulopathy prior to the onset of detectable bleeding. Interestingly, 20% of patients bitten by *E. ocellatus* in northern Cameroon were diagnosed with primary fibrinolysis, confirmed by a significant increase FDPs without other abnormalities of blood coagulation parameters [10]. Secondary fibrinolysis was observed in a further 40% of patients belonging to the same group, marked by significant increase of the FDPs and at least two other defective clotting factors (afibrinogenemia, thrombocytopenia or prolonged prothrombin time). There may have also been an association between both primary and secondary fibrinolysis in these patients.

Due to the considerable differences observed between the two reading times, clinically significant changes in hemostasis (initial resolution, secondary resumption and final resolution of coagulopathy) could be easily missed or misinterpreted if the WBCT was evaluated solely at 20 or 30 min. Based on the rapid formation and degradation of clots observed in the first 15 min after sample collection, it appears that the 20-min marker represents the earliest accurate reading time for WBCT evaluation. This period of formation and degradation of multiple clots during the first 15 or so minutes after collection represents another potential source of error in WBCT interpretation.

One author (JB) observed busy nurses and trained laboratory technicians in multiple healthcare centers as they set aside WBCT samples after collection and returned 5–10 min later to check for clot formation. If a clot was present, the test was reported as negative (no coagulopathy) and the sample was disposed of. This error was encountered on a number of occasions while conducting snakebite training for rural healthcare workers in both Benin and Kenya. The healthcare staff was under the impression that the formation of an apparently solid clot *within* 20 min constituted a negative WBCT (no coagulopathy). This could occur as a result of the large number of samples requiring processing and interpretation at a given time, or as a misunderstanding due to the fact that many of the standard coagulation tests are evaluated on the basis of time-to-coagulation rather than at a specific reading time.

Our observations during this study suggest that early WBCT interpretation, particularly in the first 15 min after collection, has a high likelihood of generating false negative results due to the repeated formation and degradation of transient clots that occurs during this time. This error may have contributed to the high number of false negatives and 40% sensitivity reported in a 2013 study on patients envenomed by Russell’s vipers [16]. In that occasion, WBCT20 testing was carried out on the wards by clinical staff without training or supervision from study investigators [16].

A recent study by the same investigators found much higher sensitivity (82%) and specificity (98%) when the WBCT20 was standardized and performed exclusively by healthcare workers trained to conduct the test correctly [11]. It is worth noting, however, that there were still 14 false positives and 14 false negatives recorded in the tested 79 patients with VICC [11]. One study on diagnosis of green pit viper (*Cryptelytops* sp.) envenomations found a sensitivity of 85.7% and specificity of 95.8% for the WBCT20 when the fibrinogen level was less than 1.0 g/L [4]. Research conducted in Papua New Guinea on reliability of the WBCT20 under different conditions reported a positive predictive value of 89.7%, negative predictive value of 93.5%, sensitivity of 92.9%, and specificity of 90.6% when fibrinogen concentrations were lower than 0.5 g/L in patients suffering from taipan (*Oxyuranus scutellatus*) snakebite [17]. All of these results indicate the need for greater standardization to ensure that tests are read precisely at the 20th and/or 30th minutes instead of within that time period, as well as the need for further research into the influence of the reading time overall. They also suggest that when performed correctly, the WBCT remains an important tool for diagnosis of VICC when costly or more complex tests of coagulation are not available.

Another issue that must be further investigated is the effect of the tube material (glass type, plastic, new, used, wide, narrow etc.) on the WBCT results. The test should be performed in glass containers to facilitate activation of the intrinsic pathway by Hageman factor (factor XII), and it is widely accepted that plastic tubes should be avoided for this reason [15]. A small 2015 study on the WBCT20 reported accurate results using soda-lime glass tubes and inaccurate results from both borosilicate glass and BD Vacutainer® glass tubes at some temperatures [17]. While it is relatively easy to selectively avoid plastic tubes, many rural hospitals and clinics in Africa clean and reuse the same glass tubes until they are broken or scratched and discarded. Healthcare facilities in rural Africa and elsewhere in the developing world often accumulate donated and purchased laboratory glassware from a multitude of sources over the years. It would be unrealistic to assume that they will have the means or the time to accurately determine the chemical composition of this glassware with the limited resources available to them on a daily basis.

Even if it were possible for all facilities to purchase only soda-lime glass tubes for these tests, the abundance of counterfeit medical supplies in West Africa nullifies the value of such an approach. We observed this issue firsthand on numerous occasions including fake antivenom composed of starches instead of proteins, new boxes of counterfeit Red-Top BD Vacutainer® tubes that were contaminated with a chemical residue, and even a shipment of grounded electrical cords missing all of the copper grounding wire except for an inch on either end to give the illusion of a genuine product. Following initial treatment in busy hospitals throughout sub-Saharan Africa, it is not uncommon for episodic surveillance of coagulation by WBCT to cease once a patient has demonstrated resolution of the hemorrhagic syndrome by H_6_, H_12_, H_24_, or H_48_ based on the assumption that permanent resolution has been achieved. The majority of resumptions we observed occurred 24 to 96 h after initial treatment at H_0_ and would not have been detected in most cases unless a consistent regimen of WBCT evaluation was maintained throughout hospitalization or if the coagulopathy was allowed to develop undetected until significant internal hemorrhage or anemia was identified symptomatically and provoked further WBCT testing.

It is possible that a nexus exists between the half-life of the antivenom, the decrease in local edema, and the recurrence of an envenomation. Venom reservoirs could theoretically become trapped in tight tissue compartments such as the tibialis. The swelling often takes 48 to 72 h before beginning to decrease, at which point the half-life of the F(ab)_2_ immunoglobulins has been exceeded. If the venom trapped in the reservoir was able to escape from the tissue compartment during this time, it could overwhelm the amount of circulating antivenom that remains and produce the recurrent envenomations observed in these cases.

The clinical significance of these findings is multifaceted. In the context of the current shortage of effective antivenom for snakebite in sub-Saharan Africa, any improvements in the efficiency of antivenom usage can save additional lives by conserving antivenom for cases in which it is required and also prevents considerable financial burden for the patient. These findings may have implications for evaluating the efficacy of new antivenoms and identifying when neutralizing capacity differs based on the composition of the venom [28].

Many prospective antivenoms for sub-Saharan Africa have been assessed using only WBCT20 or WBCT30 evaluation, but not both. If the trends observed in this study are accurate, then we would expect antivenoms assessed at 20 min to demonstrate earlier resolution of coagulopathy and later secondary resumptions of coagulopathy (or fewer resumptions overall, if the resumptions do not occur until after WBCT testing has ceased). Antivenoms assessed at 30 min would appear by comparison to be significantly less effective with longer time to restoration of normal coagulation states and greater incidence of secondary resumptions of coagulopathy. Future studies on this subject may benefit from strictly adhering to a standardized reading time (20 min, 30 min, or both) and a standardized timeline for sequential readings (both frequency and duration of WBCT testing, for example at H_3_, H_6_, H_12_, H_24_, H_48_, H_72_, and so on until the half-life of circulating antivenom has been exceeded by 24 to 48 h without a resumption of symptoms or coagulopathy).

The findings of this study may also allow for improvements in the efficiency of snakebite treatment using existing antivenoms. Had we followed the treatment protocol used in this study in conjunction with the results of WBCT30 alone, more antivenom would have been administered due to the higher number of persistent coagulopathies and transient resumptions observed. It is unknown whether the additional treatment would have benefited any of these patients, but in a small number of patients with complicated internal hemorrhage and lengthy treatment delay, the coagulopathies persisted regardless of the amount of antivenom administered and may have resulted from the physiology of the patient rather than the action of the antivenom.

Interestingly, there was a marked improvement in signs and symptoms within 24 h of the additional antivenom doses in these patients despite the persistence of coagulopathy for several more days. This may reflect an inertia in the restoration of completed depleted clotting factors in some patients. There were some instances in which WBCT30 was the first test to detect a clinically significant resumption of symptomatic coagulopathy whereas WBCT20 remained within normal range considerably longer. Interpreting the results of the test in the context of the patient is paramount. For example, WBCT testing of a patient reporting an onset of new symptoms several days after treatment may help determine whether the change in condition is due to an immune response to the antivenom or the action of some quantity of venom that is actively circulating in the bloodstream. Whether or not these trends are applicable in a broader context warrants further, large scale systematic evaluation. Until programmatic attention is given to the problem of snakebite, potentially useful tools to combat the resulting morbidity and mortality will remain under evaluated and controversial.

## Conclusion

There are few tests as readily available and informative to the clinician treating snakebites as the WBCT. It is not perfect, but it does provide a practical and functional solution where none exists. We found no indications that either reading time was superior overall. Each test appeared more or less capable of detecting different events under different circumstances than its counterpart, and neither test on its own provided an alternative to basic clinical medicine and a sound physical examination. Discrepancies in WBCT results should be interpreted together in the context of the overall clinical picture at the time of assessment.

Expanding interpretation of the WBCT to include evaluation at both 20 and 30 min may allow for a reduction in the number of false negatives or false positives during initial assessment as well as greater detection of recurrent envenomation later in the course of care. If these differences remain consistent in larger studies, it could provide a simple way to increase the tools available to medical providers for the assessment, diagnosis, treatment, and extended management of snakebite patients in sub-Saharan Africa. Though further study is needed to investigate the extent of differences between the two methods of WBCT testing, by examining both we gained a more complete view of the improvement, deterioration, and response to antivenom treatment of our patients, especially in the setting of *Echis ocellatus* envenomation. Further studies exploring the reading time and involving more complete hematological analyses are planned to confirm and explain these results.

## Abbreviations

AA: Antivipmyn® Africa (Bioclon, Mexico) polyvalent antivenom
FDPs: Fibrin degradation products
H_0_: Hour 0, initial assessment upon arrival at the hospital
H_a_: Number of hours since H_0_
VICC: Venom-induced consumption coagulopathy
WBCT: Whole blood clotting test
WBCT20 ≠ WBCT30: Discrepancies between results of WBCT20 and WBCT30
WBCT20: Whole blood clotting test with a reading time of 20 min
WBCT30: Whole blood clotting test with a reading time of 30 min
